# Supplementary material for: Increasing atmospheric dryness reduces boreal forest tree growth
Source: Nat Commun. 2023 Oct 30;14:6901. doi: 10.1038/s41467-023-42466-1 (PMC10616230; doi:10.1038/s41467-023-42466-1)
Supplement: Supplementary file 1 — Supplementary Information [file 41467_2023_42466_MOESM1_ESM.pdf]

Increasing atmospheric dryness reduces boreal forest tree growth

Supplementary Information

Supplementary Methods

In a hydrometeorological perspective, higher atmospheric dryness increases atmospheric demand for water from the land surface, and this drives an increase in evapotranspiration and thus a lowering of the available soil moisture[26]. Hence, response to atmospheric dryness can be due to direct VPD effects on conductance and indirect effects on soil moisture through VPD driving a rise of the evapotranspiration. To differentiate the two processes apart, we examined the unique contribution of VPD to the tree growth response. We implemented partial models, where goodness-of-fit for models of BAI in relation to VPD was obtained after removing the effects of soil moisture availability index (SMI). We then reported the percentage of sites for which VPD remained significant.

(A1.a) $\log\left(\mathrm{BAI}_{jk}^{t}\right) = \beta_{jk}.\log\left(BA_{jk}^{t-1}\right) + \mathrm{s}(\mathrm{age}^{t}) + \mathrm{SMI}^{t} + \mathrm{corAR1}_{jk}(\sim t \mid \mathrm{Tree}_{ID}) + \mathrm{Tree}_{ID} + \epsilon_{jkt}$

(A1.b) $\mathrm{Residuals}_{jk} = \mathrm{VPD}^{t-1} + \mathrm{VPD}^{t} + \mathrm{Tree}_{ID} + \epsilon_{jkt}$

Where i stand for tree identity, j for the species, k for the site, and t for the year. BA the basal area, BAI the basal area increment, age is the age in years, and s a cubic regression spline smoothing parameter whose degree of smoothness was determined through an iterative fitting process. Temporal autocorrelation was considered with AR1, an autoregressive term of order 1 accounting for year *t* and Tree_{ID} that is tree’s unique identifier. The significance of variables

at the 5% level was determined from *t*-test*s* in the second GAMM models (*t*-value's $p < 0.05$). The GAMM models were fitted using the mgcv R package[70].

After removing the effects of SMI, we found that most of the VPD signal was retained (Fig. S4 and Tables S2-S3). Over 27% of the sites-species combinations still had a significant relationship with VPD. VPD was mostly negatively correlated to growth, with the majority (92%) of the significant sites-species combination showing a negative relationship for VPD_t or VPD_{t-1}. These analyses suggest that stomatal sensitivity to VPD would represent the primary direct response to rising VPD.

Supplementary Figures

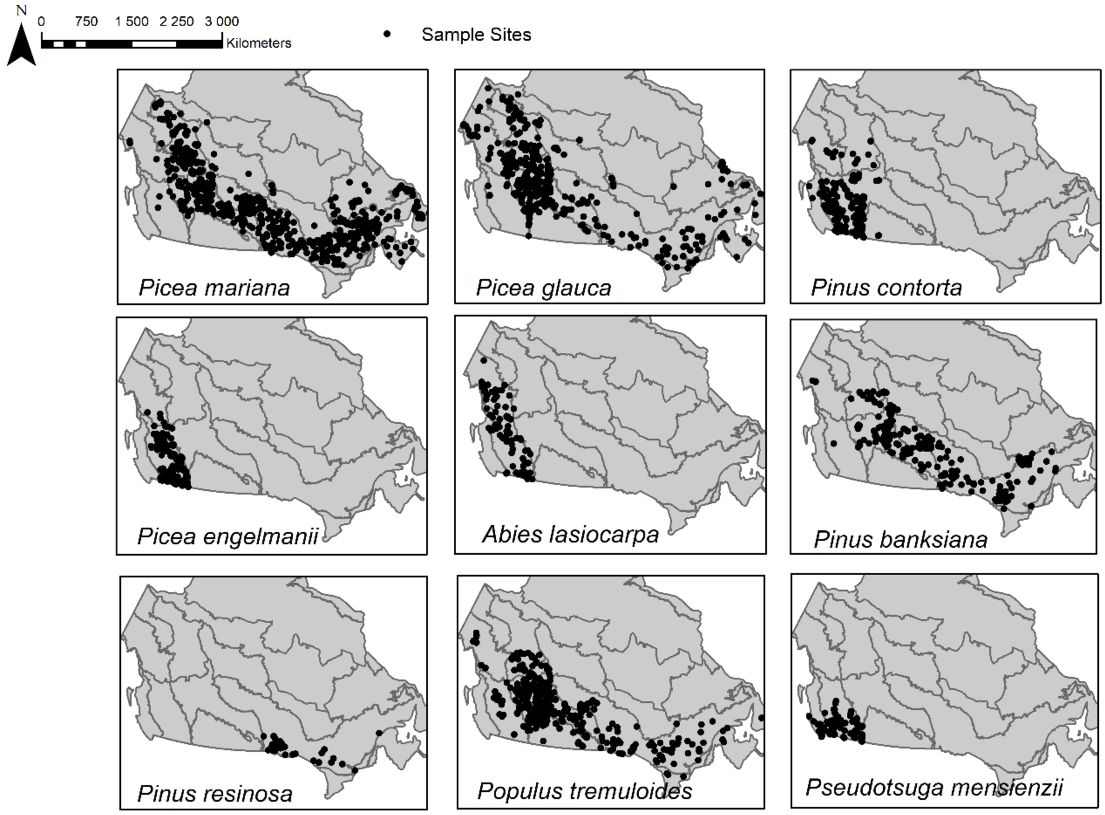

Supplementary Figure S1. Distribution of sample sites for all nine species.

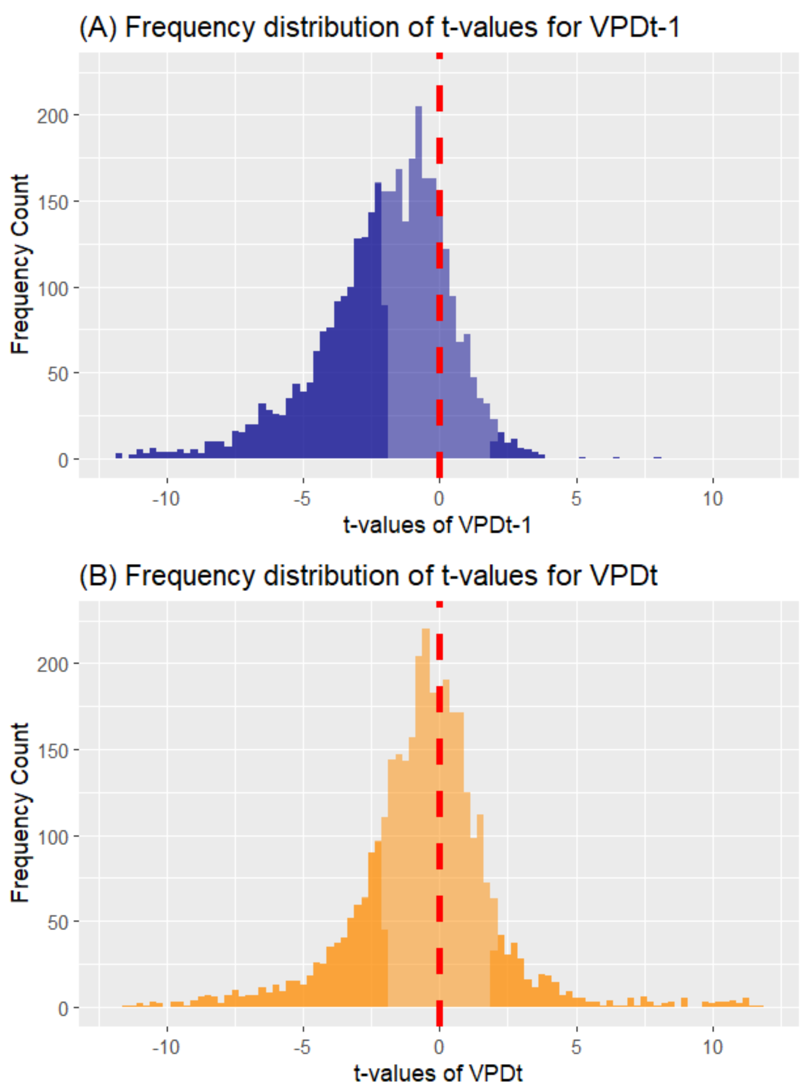

Supplementary Figure S2. Frequency distribution of t-values for the relationship between annual growth fluctuations estimated from tree rings (BAI) and summer vapour pressure deficit (VPD) of prior (t-1) and current (t) years to growth. Darker colors delineate the distribution of significant (*P*<0.05) values. Sample size: N = 3,559 species-site combinations.

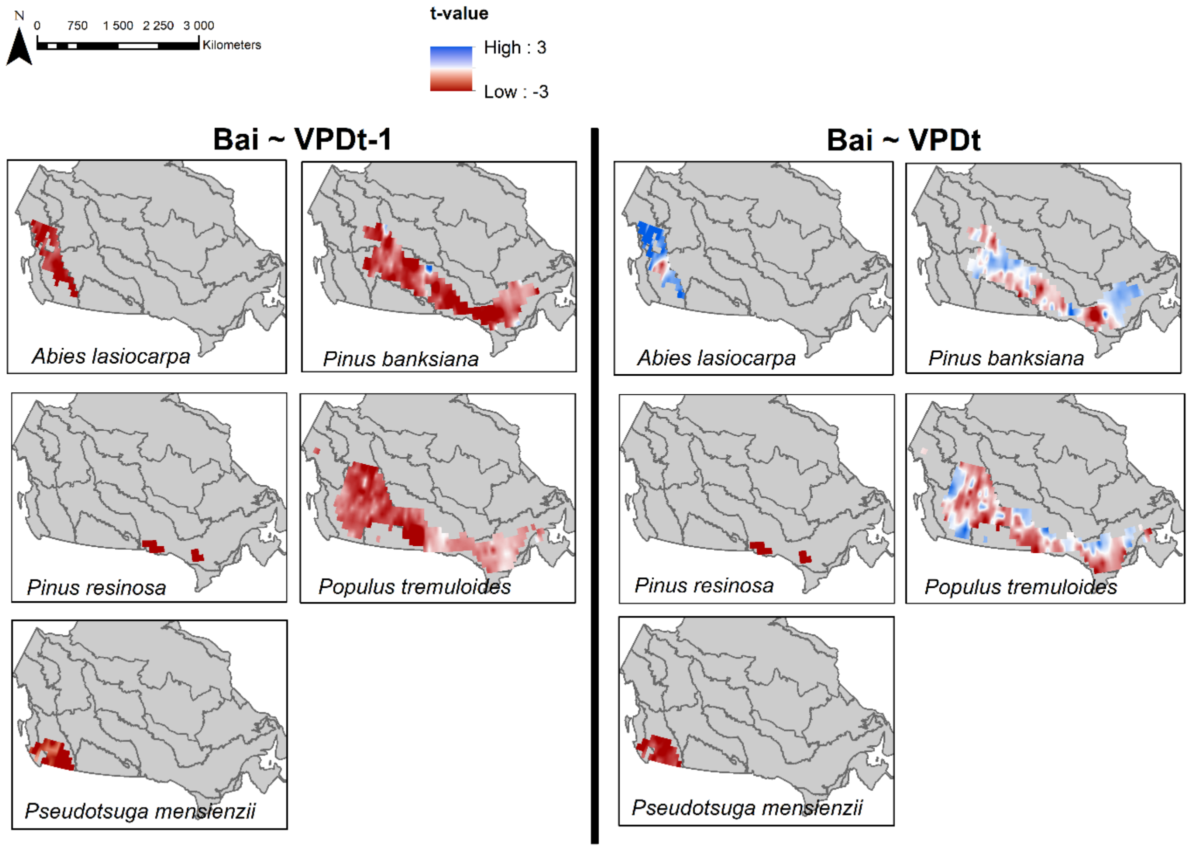

Supplementary Figure S3. Pointwise *t*-values of the regression between annual growth fluctuations estimated from tree rings (BAI) and vapour pressure deficit (VPD) for the species *Abies lasiocarpa, Pinus banksiana, Pinus resinosa, Populus tremuloides* and *Pseudotsuga mensienzii*. Maps display site-species *t*-values; a bidimensional interpolation was performed on a spatial resolution of 1 x 1 degree, using the inverse distance weighting method based on the 12 closest neighbours. Interpolations were bounded using boreal mask and species distribution area (species maps)[81,82].

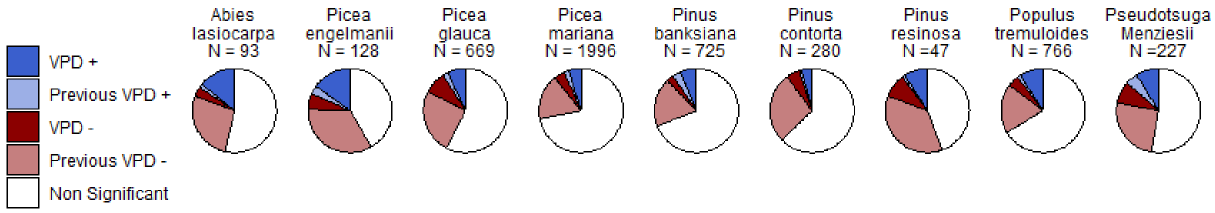

Supplementary Figure S4. Proportion of significant (*p* < 0.05), positive and negative *t*-values for VPD_t and VPD_{t-1} from the regression between annual growth and VPD for the nine tree species obtained after removing the effects of soil moisture availability index (SMI). N indicates the number of corresponding sites.

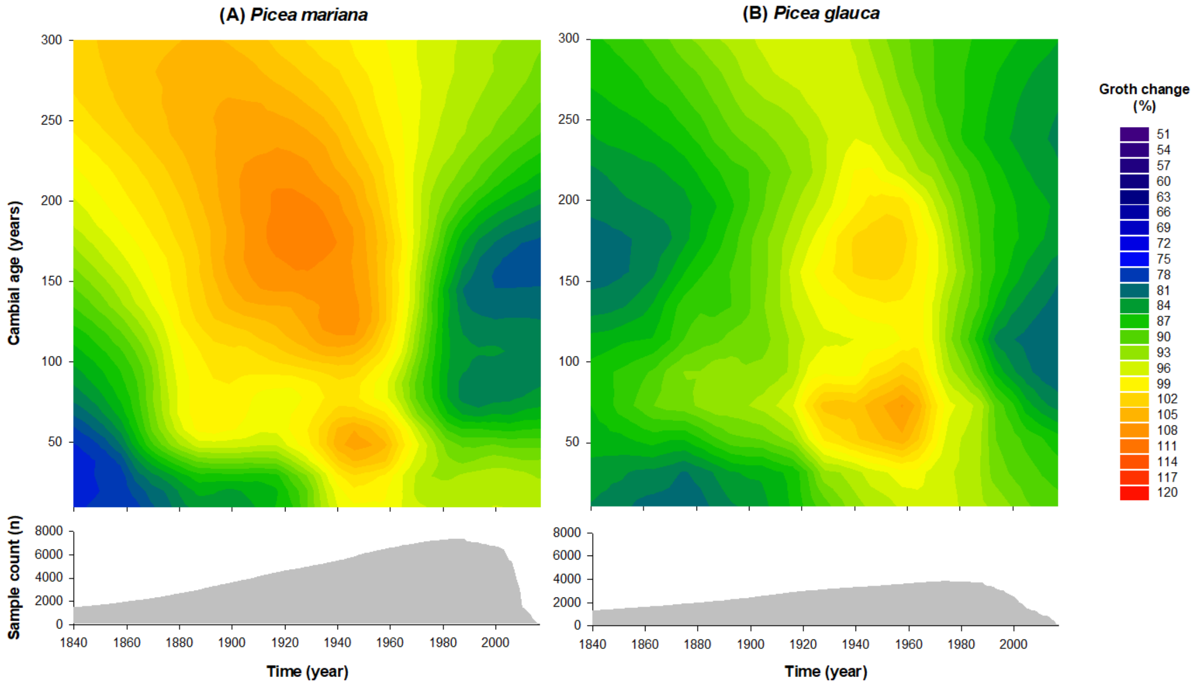

Supplementary Figure S5. Changes in the annual growth of *Picea mariana* and *Picea glauca* in the cambial age and time continuums. Annual growth changes are percent deviation from predicted values generated by the generalized additive mixed models representing the BAI variations unrelated to tree development stage (see Methods, eq. 3). Red indicates periods for a given cambial age above typical growth in the two-dimensional continuums; blue indicates periods for a given cambial age below typical growth. Contour interpolations was done using a running median smoother with the bandwidth radius set to 5% of the data. Distributions of sampled rings across years are shown by the area plots. The contour plots suggest that decreases in tree growth from 1951 to present were apparent in both species and across all cambial ages.

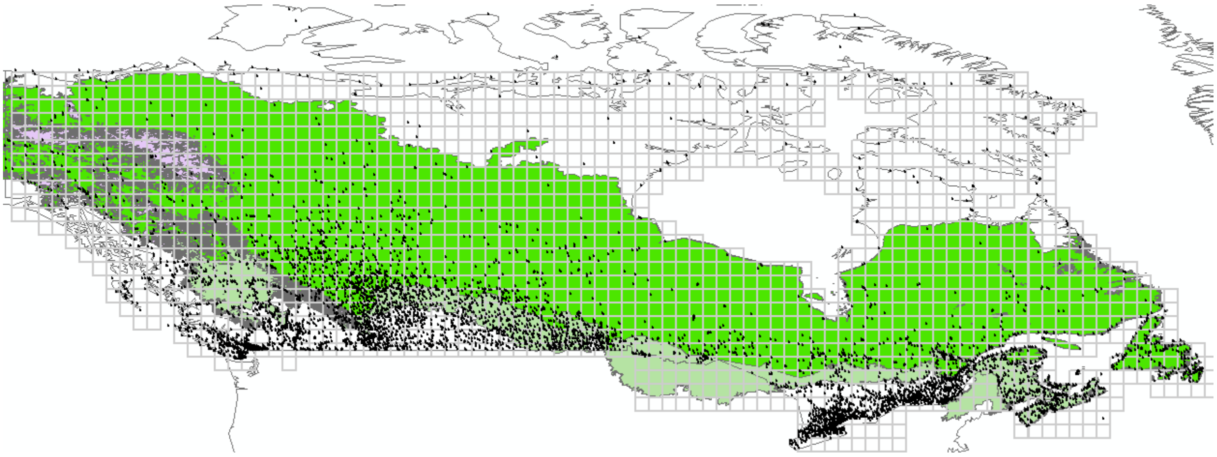

Supplemantary Figure S6. Distribution of weather stations across Canada covering in full or partly the period of 1950 to 2018. The shaded areas delineate the studied regions (green: boreal, grey: hemiboreal). For this study, weather data were resampled to a 1° x 1° grid (n = 1705 grid points) to produce homogeneous and long-term time series for establishing climate–growth relationships.

Supplementary Tabless

Supplementary Table S1. Proportion of the GAMM convergent species-site models for which VPD_{t-1} or VPD_t were significant predictors for growth. Columns detail the proportions of models for which VPD_{t-1}/ VPD_t were positively/negatively correlated to growth.

**Species**	***VPD_t or VDP_{t-1}***	***VPD_{t-1}***	***Pos. VPD_{t-1}***	***Neg. VPD_{t-1}***	***VPD_t***	***Pos. VPD_t***	***Neg. VPD_t***
Abies lasiocarpa	0.71	0.52	0.02	0.49	0.45	0.39	0.06
Picea engelmanii	0.80	0.67	0.02	0.66	0.61	0.48	0.12
Picea glauca	0.61	0.54	0.02	0.52	0.36	0.08	0.28
Picea mariana	0.24	0.20	0.01	0.19	0.10	0.03	0.06
Pinus banksiana	0.28	0.24	0.01	0.22	0.11	0.06	0.05
Pinus contorta	0.68	0.61	0.02	0.59	0.35	0.09	0.26
Pinus resinosa	0.85	0.74	0.00	0.74	0.83	0.04	0.79
Populus tremuloides	0.48	0.34	0.01	0.33	0.29	0.08	0.21
Pseudotsuga menziesii	0.67	0.53	0.01	0.52	0.48	0.01	0.47

Supplementary Table S2. Proportion of the GAMM convergent species-site models for which VPD_{t-1} or VPD_t were significant predictors for growth, obtained after removing the effects of soil moisture availability index (SMI). Columns detail the proportions of models for which VPD_{t-1}/VPD_t were positively/negatively correlated to growth.

**Species**	***VPD_t or VPD_{t-1}***	***VPD_{t-1}***	***Pos. VPD_{t-1}***	***Neg. VPD_{t-1}***	***VPD_t***	***Pos. VPD_t***	***Neg. VPD_t***
Abies lasiocarpa	0.54	0.41	0.02	0.39	0.26	0.20	0.05
Picea engelmanii	0.68	0.62	0.05	0.56	0.35	0.25	0.10
Picea glauca	0.39	0.32	0.02	0.29	0.18	0.08	0.10
Picea mariana	0.15	0.12	0.01	0.11	0.05	0.03	0.03
Pinus banksiana	0.20	0.16	0.02	0.14	0.07	0.05	0.02
Pinus contorta	0.46	0.40	0.01	0.39	0.13	0.06	0.07
Pinus resinosa	0.79	0.70	0.02	0.68	0.34	0.17	0.17
Populus tremuloides	0.30	0.21	0.01	0.19	0.14	0.09	0.04
Pseudotsuga menziesii	0.51	0.44	0.07	0.37	0.25	0.13	0.12

Supplementary Table S3. Statistics of species-specific Generalized Additive Mixed Models (GAMM). The r-squared (r2) and mean squared error (mse) are computed from the square of the Pearson correlation between observed and predicted log-transformed BAI (LBAI) and power-scaled BAI (BAI). The count of sampled rings, trees and sites for each GAMM is given by No rings, No trees and No sites. Mean series length (years) is indicated by avg series.

Species	***No rings***	***No sites***	***No trees***	***avg series***	***LBAI r2***	***LBAI mse***	***BAI r2***	***BAI mse***
Abies lasiocarpa	139400	89	923	151	0.889	0.130	0.812	2.403
Pseudotsuga menziesii	352003	227	2754	128	0.766	0.295	0.714	29.786
Picea engelmannii	318373	122	1632	195	0.902	0.137	0.822	8.785
Picea glauca	612695	642	4522	135	0.908	0.208	0.837	127.344
Picea mariana	998924	1937	9481	105	0.858	0.145	0.687	1.256
Pinus banksiana	260364	573	3586	73	0.835	0.210	0.759	3.956
Pinus contorta	270095	268	2859	94	0.783	0.172	0.721	3.344
Pinus resinosa	102927	47	868	119	0.773	0.239	0.771	36.620
Populus tremuloides	194580	706	3164	61	0.633	0.351	0.589	15.116
