# Supplementary material for: Increasing atmospheric dryness reduces boreal forest tree growth
Source: Nat Commun. 2023 Oct 30;14:6901. doi: 10.1038/s41467-023-42466-1 (PMC10616230; doi:10.1038/s41467-023-42466-1)
Supplement: Supplementary file 3 — Reporting Summary [file 41467_2023_42466_MOESM3_ESM.pdf]

nature portfolio

Corresponding author(s): Ariane Mirabel

Last updated by author(s): 2023-10-05

Reporting Summary

Nature Portfolio wishes to improve the reproducibility of the work that we publish. This form provides structure for consistency and transparency in reporting. For further information on Nature Portfolio policies, see our Editorial Policies and the Editorial Policy Checklist.

Statistics

For all statistical analyses, confirm that the following items are present in the figure legend, table legend, main text, or Methods section.

n/a	Confirmed	
☐	☒	The exact sample size (n) for each experimental group/condition, given as a discrete number and unit of measurement
☐	☒	A statement on whether measurements were taken from distinct samples or whether the same sample was measured repeatedly
☐	☒	The statistical test(s) used AND whether they are one- or two-sided<br>*Only common tests should be described solely by name; describe more complex techniques in the Methods section.*
☐	☒	A description of all covariates tested
☐	☒	A description of any assumptions or corrections, such as tests of normality and adjustment for multiple comparisons
☐	☒	A full description of the statistical parameters including central tendency (e.g. means) or other basic estimates (e.g. regression coefficient) AND variation (e.g. standard deviation) or associated estimates of uncertainty (e.g. confidence intervals)
☐	☒	For null hypothesis testing, the test statistic (e.g. F, t, r) with confidence intervals, effect sizes, degrees of freedom and P value noted<br>*Give P values as exact values whenever suitable.*
☒	☐	For Bayesian analysis, information on the choice of priors and Markov chain Monte Carlo settings
☐	☒	For hierarchical and complex designs, identification of the appropriate level for tests and full reporting of outcomes
☐	☒	Estimates of effect sizes (e.g. Cohen's d, Pearson's r), indicating how they were calculated

Our web collection on statistics for biologists contains articles on many of the points above.

Software and code

Policy information about availability of computer code

Data collection	Daily weather data were retrieved from Environment and Climate Change Canada’s web portal and processed using BioSIM v10.3 software (https://cfs.nrcan.gc.ca/projects/133).
Data analysis	- The growth model was fitted using 'mgcv' R package v1.8.41<br>-The running and interpretation of the random forest (RF) were performed using 'randomForest' v4.7.1.1 and 'randomForestExplainer' v0.10.1 R packages.<br>- Autocorrelation estimates of Moran's I were computed using the moran.test function in R<br>- Least squares linear regressions were conducted using the Sigmaplot v14 software<br>- Correlation and bootstrapping were achieved using the software provided by Mudelsee et al. (2007) (article referenced in text)<br>- The R scripts and codes used to perform the analysis are available in the following GitHub repository: https://github.com/ArianeMirabel/Dendrochronology

For manuscripts utilizing custom algorithms or software that are central to the research but not yet described in published literature, software must be made available to editors and reviewers. We strongly encourage code deposition in a community repository (e.g. GitHub). See the Nature Portfolio guidelines for submitting code & software for further information.

All manuscripts must include a data availability statement. This statement should provide the following information, where applicable:

- Accession codes, unique identifiers, or web links for publicly available datasets
- A description of any restrictions on data availability
- For clinical datasets or third party data, please ensure that the statement adheres to our policy

Data

Policy information about availability of data

Weather data that support the finding of this sutdy are freely accessible through Environment and Cimate Change Canada's portal (https:/climate.weather.gc.ca/) and the BioSIM server (https://cfs.nrcan.gc.ca/projects/133). Tree-ring datasets were deposited in the Natural Resources Canada TreeSource repository https:// treesource.rncan.gc.ca/en. Restrictions may apply to the availability of third-party data (contact details are included in the TreeSource repository).
The data that support the plots within this paper and other findings of this study are deposited on the FigShare repository 10.6084/m9.figshare.24260554.
The analysis and plotting scripts are available in the GitHub repository https://github.com/ArianeMirabel/Dendrochronology/, DOI: 10.5281/zenodo.8410445

Research involving human participants, their data, or biological material

Policy information about studies with human participants or human data. See also policy information about sex, gender (identity/presentation), and sexual orientation and race, ethnicity and racism.

Reporting on sex and gender

Use the terms sex (biological attribute) and gender (shaped by social and cultural circumstances) carefully in order to avoid confusing both terms. Indicate if findings apply to only one sex or gender; describe whether sex and gender were considered in study design; whether sex and/or gender was determined based on self-reporting or assigned and methods used.
Provide in the source data disaggregated sex and gender data, where this information has been collected, and if consent has been obtained for sharing of individual-level data; provide overall numbers in this Reporting Summary. Please state if this information has not been collected.
Report sex- and gender-based analyses where performed, justify reasons for lack of sex- and gender-based analysis.

Reporting on race, ethnicity, or other socially relevant groupings

Please specify the socially constructed or socially relevant categorization variable(s) used in your manuscript and explain why they were used. Please note that such variables should not be used as proxies for other socially constructed/relevant variables (for example, race or ethnicity should not be used as a proxy for socioeconomic status).
Provide clear definitions of the relevant terms used, how they were provided (by the participants/respondents, the researchers, or third parties), and the method(s) used to classify people into the different categories (e.g. self-report, census or administrative data, social media data, etc.)
Please provide details about how you controlled for confounding variables in your analyses.

Population characteristics

Describe the covariate-relevant population characteristics of the human research participants (e.g. age, genotypic information, past and current diagnosis and treatment categories). If you filled out the behavioural & social sciences study design questions and have nothing to add here, write "See above."

Recruitment

Describe how participants were recruited. Outline any potential self-selection bias or other biases that may be present and how these are likely to impact results.

Ethics oversight

Identify the organization(s) that approved the study protocol.

Note that full information on the approval of the study protocol must also be provided in the manuscript.

Field-specific reporting

Please select the one below that is the best fit for your research. If you are not sure, read the appropriate sections before making your selection.

☐ Life sciences ☐ Behavioural & social sciences ☒ Ecological, evolutionary & environmental sciences

For a reference copy of the document with all sections, see nature.com/documents/nr-reporting-summary-flat.pdf

Ecological, evolutionary & environmental sciences study design

All studies must disclose on these points even when the disclosure is negative.

Study description

We assess boreal forest responses to changes in atmospheric VPD using a well-replicated tree-ring network covering Canada's forests over the period 1951-2018. Using mixed-effects models, we quantified annual growth changes as a function of atmospheric VPD of the prior and current year of tree-ring formation. The growth-VPD relationships enabled mapping of spatially-explicit VPD responses across Canada's boreal zone. We then explored the main drivers of differential growth responses to VPD, including species, local precipitation and temperature, elevation, and tree age and size using Random Forest algorithms. Finally, we determined how VPD and growth were changing over time for the most responsive species.

Research sample

Analyses were conducted on nine dominant tree species (seven genera) of Canada's forests. These nine species represent 80% of the samples (32,189 trees) on the CFS-TRendD repository : Picea mariana (black spruce; 25% of the dataset with 1.0 e4 trees sampled), Picea glauca (white spruce; 12%, 4.7 e3 trees), Pinus banksiana (jack pine; 11%, 4 4 e3 trees), Populus tremuloides (trembling aspen; 10%, 3.9 e3 trees), Pinus contorta (lodgepole pine; 7%, 2.9 e3 trees), Pseudotsuga menziesii (Douglas fir; 7%, 2.8 e3 trees), Picea engelmannii (Engelmann spruce; 4%, 1.7 e3 trees), Abies lasiocarpa (subalpine fir; 2%, 9.3 e2 trees), and Pinus resinosa (red pine; 2%, 8.7e2 trees). All of the nine species occur in Canadian boreal/hemi-boreal forests. Half of the sites included two or three sampled trees, and 83% of the sites included a single species. Fifty percent of the trees displayed between 40 and 90 measured tree rings (i.e 25th and 75th percentiles of the age distribution).
The primary national-scale dataset in CFS-TRenD 1.0 is increment cores sampled since 2001 during the establishment of Canada's [illegible]

National Forest Inventory (NFI). Other important data are more regional and include data from a network of permanent sample plots established by the Alberta Biodiversity Monitoring Institute (ABMI), a network of temporary sample plots established by the Ministère des Ressources Naturelles et de la Faune du Québec (MFFPQ; Programme d'inventaire écoforestier nordique), and the Climate Impacts on Productivity and Health of Aspen (CIPHA) network. Additional contributions to CFS-TRenD are from smaller-scale projects carried out by individual researchers, and data extracted from the International Tree-Ring Data Bank (ITRDB). The complete list of contributions is detailed in Girardin et al. (2021).

Daily maximum, minimum temperature, precipitation, and relative humidity were obtained for the period from 1950 to 2018. Using daily minimum and maximum temperature as well as precipitation data, we employed the BioSIM software to calculate the daily vapor pressure deficit following the approach described in Allen et al. (1998). For dewpoint temperature calculation, BioSIM utilizes the methodology outlined in Kimball et al. (1997) and leverages daily temperature and precipitation data. The soil moisture index (SMI) was estimated in BioSIM using the quadratic-plus-linear (QL) formulation procedure described in Régnières et al. (2014).

(All articles are referenced in main text)

Sampling strategy

Weather data were interpolated to a 1x1 degree grid using BioSIM v10.3 software. VPD and SMI data were averaged for the summer season (June to August) by year, and attributed to the closest tree-ring sampling sites' grid point. In addition, long-term means of annual climatologies (1951-2018) were also computed for each site (mean annual January to December temperature, MAT; mean annual January to December sums of precipitation, MAP; and mean summer soil moisture index, SMI).

The choice of the selected species was made so to maintain a sampling density that balances locals variations in growth across sampling sites within regions, and detects growth sensitivity to climate at the regional scale. We selected species with a minimum of 50 species-site combinations, and a visual check was made to ensure a homogeneous repartition over individual species distribution area.

Data collection

Annual tree-ring width data were retrieved from the Canadian Forest Service Tree-Ring repository (CFS-TRenD 1.031), developed with the goal of combining data from different sources and making them available in a consistent format for large-scale analyses. CFS-TRenD is a growing data repository that represents significant investments by multiple researchers. Samples contained therein are the result of multiple sampling designs, including forest inventory tree-ring collections and targeted sampling collections of old trees on ecologically marginal sites. The database is cleaned and organized by data custodians to create a cohesive structure of quality-controlled and corrected data from multiple sources. CFS-TRenD is a central repository, conveniently structured following an internationally used workflow akin to ETL (extract, transform, load) processes. Tree-ring data are received directly from contributors or extracted from online resources (i.e., ITRDB) and transformed (deletion of duplicate records, data cleaning, key restructuring, data validation, format correction) before being loaded into the repository.

Timing and spatial scale

Analyses cover Canada's boreal/hemi-boreal zones over the period of 1951-2018

Data exclusions

We excluded Abies balsamea (balsam fir, mostly dominant in the eastern ecozones) because its growths dynamics are strongly influenced by spruce budworm (Choristoneura fumiferana) defoliation.

Reproducibility

All analyses are reproducible. Analyses were reproduced by a statistician of the Canadian Forest Service using methodological approaches that were different from those taken here, and results were found to be reproducible. The software for analysis are common, and the statistical analysis documented.

Randomization

Randomization was not relevant as no experiment was conducted.

Blinding

Blinding was not relevant in our study as tree-ring sampling protocols are standardize and homogeneous among regions and along time. No different groups were made during data analysis, and similar methods were applied to the whole dataset.

Did the study involve field work? ☐ Yes ☒ No

Reporting for specific materials, systems and methods

We require information from authors about some types of materials, experimental systems and methods used in many studies. Here, indicate whether each material, system or method listed is relevant to your study. If you are not sure if a list item applies to your research, read the appropriate section before selecting a response.

Materials & experimental systems

n/a	Involved in the study
☒	☐ Antibodies
☒	☐ Eukaryotic cell lines
☒	☐ Palaeontology and archaeology
☒	☐ Animals and other organisms
☒	☐ Clinical data
☒	☐ Dual use research of concern
☒	☐ Plants

Methods

n/a	Involved in the study
☒	☐ ChIP-seq
☒	☐ Flow cytometry
☒	☐ MRI-based neuroimaging
